# Supplementary material for: Levels of Vitamin D and Expression of the Vitamin D Receptor in Relation to Breast Cancer Risk and Survival
Source: Nutrients. 2022 Aug 16;14(16):3353. doi: 10.3390/nu14163353 (PMC9414444; doi:10.3390/nu14163353)
Supplement: Supplementary file 1 [file nutrients-14-03353-s001.zip › nutrients-1784145-supplementary.pdf]

Supplementary Table S1: Distribution of tumor characteristics in relation to tertile of vitamin D

	All<br>n=912				
Tumor in tissue microarray<br>number		Yes<br>718			No<br>194
Nuclear VDR assessable<br>number		Yes<br>678			No<br>40
Vitamin D level available<br>number		Yes<br>497			No<br>181
Tertile of vitamin D<br>number		1st Low<br>169	2nd Medium<br>164	3rd High<br>164	 415
Factor	n (%) or	n (%) or	n (%) or	n (%) or	n (%) or
Season of diagnosis					
Winter	241 (26.4)	43 (25.4)	48 (29.3)	38 (23.2)	112 (27)
Spring	221 (24.2)	48 (28.4)	34 (20.7)	41 (25.0)	98 (23.6)
Summer	186 (20.4)	34 (20.1)	32 (19.5)	25 (15.2)	95 (22.9)
Fall	264 (28.9)	44 (26.0)	50 (30.5)	60 (36.6)	110 (26.5)
Tumor size					
1-10 mm	229 (25.8)	29 (17.3)	38 (23.3)	40 (24.5)	121 (30.8)
11-20 mm	409 (46.1)	83 (49.4)	78 (47.9)	74 (45.4)	174 (44.3)
≥21 mm	250 (28.2)	56 (33.3)	47 (28.8)	49 (30.1)	98 (24.9)
Unknown	24	1	1	1	22
Lymph node status					
Positive	262 (31.9)	59 (36.4)	48 (32.4)	55 (35.9)	100 (28.1)
Negative	559 (68.1)	103 (63.6)	100 (67.6)	98 (64.1)	256 (71.9)
Unknown	91	7	16	11	59
Nottingham grade					
I	227 (27.2)	37 (22.3)	47 (29.7)	37 (23.3)	106 (30.1)
II	393 (47.0)	81 (48.8)	69 (43.7)	80 (50.3)	162 (46)
III	216 (25.8)	48 (28.9)	42 (26.6)	42 (26.4)	84 (23.9)
Unknown	76	3	6	5	63
Histological type					
Ductal	596 (70.9)	125 (75.3)	123 (76.4)	102 (64.2)	245 (69.2)
Lobular	166 (19.7)	34 (20.5)	24 (14.9)	44 (27.7)	64 (18.1)
Other/mixed	79 (9.4)	7 (4.2)	14 (18.7)	13 (8.2)	45 (12.7)
Unknown	71	3	3	5	61
ER status					
neg (0-10%)	84 (10.8)	21 (13.7)	19 (12.6)	20 (13.3)	24 (7.4)
pos (>10%)	694 (89.2)	132 (86.3)	132 (87.4)	130 (86.7)	299 (92.6)
Unknown	134	16	13	14	92
PgR status					
neg (0-10%)	311 (41.7)	70 (49.3)	69 (46.6)	74 (52.1)	97 (31.0)
pos (>10%)	435 (58.3)	72 (50.7)	79 (53.4)	68 (47.9)	216 (69.0)
Unknown	166	27	16	22	102
HER2					
neg	646 (90.9)	122 (87.8)	125 (93.3)	121 (91.0)	277 (91.1)
pos	65 (9.1)	17 (12.2)	9 (6.7)	12 (9.0)	27 (8.9)
Unknown	201	30	30	31	111
Ki67					
Low	258 (40.6)	50 (34.7)	50 (36.0)	59 (46.1)	99 (44.6)
Intermediate	198 (31.2)	50 (34.7)	52 (37.4)	36 (28.1)	58 (26.1)
High	179 (28.2)	44 (30.6)	37 (26.6)	33 (25.8)	65 (29.3)
Unknown	277	25	25	36	193
Molecular subtypes					
Luminal A-like	350 (55.6)	86 (56.2)	93 (57.8)	85 (57.1)	141 (56.9)
Luminal B-like	158 (25.1)	30 (19.6)	42 (26.1)	39 (24.0)	64 (25.8)
HER2 positive	65 (10.3)	18 (11.8)	14 (8.7)	12 (9.5)	27 (10.9)
Triple negative	56 (8.9)	19 (12.4)	12 (7.5)	12 (9.3)	16 (6.5)
Unknown	283	16	3	16	167
Nuclear VDR					
Negative (0-10%)	125 (18.4)	36 (21.3)	35 (21.3)	23 (14.0)	31 (17.1)
Positive (11-100%)	553 (81.6)	133 (78.7)	129 (78.7)	141 (86.0)	150 (82.9)
Unknown	234	0	0	0	234

Percentages does not include missing categories.

Supplementary Table S2: Distribution of treatment factors in relation to tertile of vitamin D

	All<br>n=912				
Tumor in tissue microarray<br>number			Yes<br>718		No<br>194
Nuclear VDR assessable<br>number			Yes<br>678		No<br>40
Vitamin D level available<br>number			Yes<br>497		No<br>181
Tertile of vitamin D<br>number		1st Low<br>169	2nd Medium<br>164	3rd High<br>164	 415
Factor	n (%)	n (%)	n (%)	n (%)	n (%)
Type of surgery					
Mastectomy	363 (41.7)	80 (47.3)	65 (40.4)	70 (42.9)	149 (39.4)
Partial mastectomy	505 (58.0)	88 (52.1)	96 (59.6)	93 (57.1)	227 (60.1)
Local excision/biopsy	3 (0.3)	1 (0.6)	0 (0.0)	0 (0.0)	2 (0.5)
Unknown	41	0	3	1	37
Axillary surgery					
No axillary dissection	80 (9.2)	7 (4.1)	16 (9.9)	11 (6.8)	45 (11.8)
Axillary dissection	506 (58.0)	118 (69.8)	90 (55.6)	111 (68.9)	188 (49.5)
Sentinel node biopsy	285 (32.7)	44 (26.0)	55 (34.0)	39 (24.2)	147 (38.7)
Singular node biopsy	1 (0.1)	0 (0.0)	1 (0.6)	0 (0.0)	0 (0.0)
Unknown	40	0	2	3	35
Planned adjuvant:					
Endocrine therapy					
No	410 (46.6)	80 (48.2)	70 (43.8)	74 (45.4)	185 (47.6)
Yes	469 (53.4)	86 (51.8)	90 (56.3)	89 (54.6)	204 (52.4)
Unknown	33	3	164	1	26
Chemotherapy					
No	698 (84.7)	127 (84.1)	125 (84.5)	126 (83.4)	319 (85.5)
Yes	126 (15.3)	24 (15.9)	23 (15.5)	25 (16.6)	54 (14.5)
Unknown	88	18	16	13	42
Radiotherapy					
No	327 (39.6)	49 (32.2)	62 (41.6)	56 (37.1)	159 (42.6)
Yes	499 (60.4)	103 (67.8)	87 (58.4)	95 (62.9)	214 (57.4)
Unknown	86	17	15	13	42

Percentages does not include missing categories.
